# Comparison of assessment scores for fatigue between multidimensional fatigue inventory (MFI-K) and modified chalder fatigue scale (mKCFQ)

**DOI:** 10.1186/s12967-021-03219-0

**Published:** 2022-01-03

**Authors:** Eun-Jin Lim, Chang-Gue Son

**Affiliations:** 1 Department of Integrative Medicine, Graduate School of Integrative Medicine, CHA University, 335 Pangyo-ro, Sungnam-si, Bundang-gu, 13488 Gyeonggi-do Korea; 2 Department of Korean Medicine, Institute of Bioscience and Integrative Medicine, Daejeon University, 62 Daehak-ro, Dong-gu, Daejeon, 300-716 Republic of Korea

**Keywords:** Fatigue severity scale, Multidimensional fatigue inventory, Chalder fatigue scale, Chronic fatigue, Questionnaire

## Abstract

**Background:**

Because of the absence of biological parameters for fatigue, appropriate instruments for assessing the degree of fatigue are important in the diagnosis and management of people complaining of fatigue-like symptoms. This study statistically analyzed the fatigue scores from two typical questionnaire-based instruments: the Korean version of the Multidimensional Fatigue Inventory (MFI-K) and the modified Chalder Fatigue Scale (mKCFQ).

**Methods:**

Seventy participants (males *n*  = 40, females *n*  = 30, median age 48 years old, range of 25–67) were grouped into three groups (‘mild’  = 20, ‘moderate’  = 42, and ‘severe’  = 8) according to self-reported fatigue levels using a 7-point Likert scale. The similarities and differences between two instrument-derived scores were analyzed using correlations (*r*) and multidimensional scaling (MDS).

**Results:**

The total scores of the two assessments were significantly correlated (*r*  = 75%, *p*  < 0.001), as were the subscores (‘Total Physical fatigue’: *r*  = 76%, *p*  < 0.001, ‘Total Mental fatigue’: *r*  = 56%, *p*  < 0.001). Relative overestimation of the MFI-K (45.8 ± 11.3) compared to the mKCFQ (36.1 ± 16.2) was observed, which was especially prominent in the ‘mild’ group. The scores of the three groups were more easily distinguished by the mKCFQ than by the MFI-K. In terms of the five dimension scores, we found a higher correlation of the two assessments for ‘general fatigue’ (*r*  = 79%,* p*  < 0.001) and ‘physical fatigue’ (*r*  = 66%, *p*  < 0.001) than for the reductions in ‘motivation’ (*r*  = 41%,* p*  < 0.01) and ‘activity’ (*r*  = 26%,* p*  > 0.05).

**Conclusions:**

Our results may indicate the usefulness of the two instruments, especially for the physical symptoms of fatigue (‘general’ and ‘physical’ fatigue). Furthermore, the MFI-K may be useful for conditions of moderate-to-severe fatigue, such as chronic fatigue syndrome, but the mKCFQ may be useful for all spectra of fatigue, including in subhealthy people.

**Supplementary Information:**

The online version contains supplementary material available at 10.1186/s12967-021-03219-0.

## Background

Fatigue is the state of weariness that may result from excessive physical and mental effort and psychological distress [1]. In general, fatigue is classified as acute, prolonged or chronic by the duration of the symptom, and chronic fatigue (fatigue  > 6 months) can be considered a medical problem [2]. Among types of chronic fatigue, chronic fatigue syndrome (CFS) is the most debilitating illness, characterized by post exertional malaise (PEM), sleep disorder, cognitive dysfunction, orthostatic intolerance and a seven-fold higher suicide rate [3]. Studies have found that up to 30–50% of the general population experienced fatigue [4, 5], approximately 10% experienced chronic fatigue [6], and a recent review study reported that 1% experienced CFS [7].

On the other hand, the recognition of fatigue has been expanded to various dimensions due to the complexity of fatigue [2, 8]. Fatigue could be a physiological response as well as a disorder; however, there is no objective biological parameter to assess fatigue, which raises problems in the diagnosis and management of fatigue [9]. Accordingly, various fatigue measurement tools have been introduced to assess fatigability [2]. To date, diverse patient-reported outcome (PRO) measurements have been developed and used to assess fatigue status in clinics. Some are fatigue-nonspecific instruments, such as the 36-item Short Form Health Survey (SF-36) [10], Clinical Global Impression (CGI) [11], and Sickness Impact Profile-8 (SIP-8) [12], while fatigue-specific tools include the Checklist Individual Strength (CIS) scale [13], Chalder Fatigue Questionnaire (CFQ) [14], and Multidimensional Fatigue Inventory (MFI) [15].

We recently reviewed the trend of fatigue-assessment instrument application in clinical trials for CFS and found that both MFI and CFQ were the most commonly employed instruments [16]. These instruments reflect the clinical features of chronic fatigue divided into physical and mental domains and further into five dimensions: general, physical, mental, reduced activity, and reduced motivation [14, 15]. In fact, several clinical studies have adopted MFI and CFQ as primary assessment tools for fatigue and CFS patients [17–21]. The selection of an appropriate assessment tool is crucial in fatigue-related clinical studies, while the Korean version of the MFI (MFI-K) and modified CFQ (mKCFQ) were designed for assessing the therapeutic process of Koreans complaining of fatigue and CFS, and each of them was clinically validated [22, 23]. However, no studies have been conducted comparing the characteristics of tools, particularly the most commonly used tools: MFI and CFQ.

The present study aims to evaluate the correlations and find the similarities and differences between the two instruments in identifying those with fatigue to determine their optimal usefulness.

## Methods

### Participants

This study comparing the MFI-K and mKCFQ was conducted from September to December 2020 among people working in a university. We collected email addresses from the university emailing group system shared only for the employees. We sent an invitation email to the 250 general employees including: educational personnel, researcher, and administrative people, and asked to participate for the survey if they consider themselves having some level of fatigue. In total 70were recruited and agreed to participate (Table 1). Prior to the survey, the level of fatigue (using a 7-point Likert scale: 1 indicates ‘no fatigue or minimal’ 7 indicates ‘most severe fatigue or intolerable’) was assessed for the purpose of grouping the participants. According to the fatigue level, the participants were grouped into three categories: ‘mild’ (fatigue level: 1–2), ‘moderate’ (3–5), and ‘severe’ (6–7). Participants who might experience discomfort, who refused to participate, who were pregnant or who had a condition that may influence the results were excluded from this study.

**Table 1:** Demographics of the participants

Participants	Group^a^			Total
	Mild	Moderate	Severe	
Total, *n*	20	42	8	70
Median age (range)	54 (35–66)	47 (30–67)	43 (25–51)	48 (25–67)
Mean BMI	26.0 ± 4.5	25.6 ± 4.8	20.4 ± 1.6	25.2 ± 5.2
Male, *n*	15	22	3	40
Median age (range)	56 (35–66)	48 (32–64)	44 (42–50)	50 (32–66)
Mean BMI	31.0 ± 4.1	25.8 ± 0.1	26.0 ± 1.2	27.4 ± 3.1
Female, *n*	5	22	5	30
Median age (range)	48 (43–56)	45 (30–67)	33 (25–51)	44 (25–67)
Mean BMI	24.3 ± 3.1	25.5 ± 5.3	20.4 ± 1.6	23.9 ± 4.3

*BMI* body mass index

^a^Grouped by fatigue level (7-point Likert scale: mild 1–2, moderate 3–5, severe 6–7)

### Study instruments

The MFI is an instrument with 20 questions (5-point Likert scale, 1 =  ‘agree’ to 5 =  ‘disagree’ for the positive and negative questions) assessing five dimensions of fatigue that are grouped into two parts: total physical fatigue (general, physical fatigue, reduced activity) and total mental fatigue (mental fatigue and reduced motivation). This instrument is designed with positive and negative questions to increase the reliability of the responses. In this study, we used the Korean version (MFI-K, Additional file 1: Table S1), which was validated with 595 participants experiencing fatigue in 2018 [22].

The CFQ is a PRO-based instrument composed of 11 questions (7 items for ‘physical fatigue’ and 4 items for ‘mental fatigue’) to assess the fatigue level based on a comparison with the “usual” status on a 4-point Likert scale (“Less than usual”, “No more than usual”, “More than usual”, and “Much more than usual”) [14]. The Korean version of the CFQ (K-CFQ) was validated with healthy participants in 2018 [24], and we used the modified Korean version (mKCFQ, Additional file 1: Table S1) in this study. To resolve the difficulty of assessment based on the comparison with the “usual” status, the mKCFQ was adapted to a 10-point Likert scale (0 =  ‘not at all’ to 9 =  ‘unbearably severe condition’), and its reliability and validity were confirmed with 97 CFS participants [23].

### Analysis of data

For the consistent comparisons of the two instruments, the mKCFQ score (0–9 points for each of the 11 questions, maximum 99 points) was converted to a 100-point scale, the same as the MFI-K score (5 points each of the 20 questions, maximum 100 points). That is, the total score of each dimension of the mKCFQ (maximum 18 points for general, mental, reduced activity and motivation and 27 points for physical fatigue) was reformulated according to the MFI-K score, which was scored on a 20-point scale on each of the five dimensions (Additional file 1: Table S1). We compared and analyzed the data based on the five dimensions of fatigue.

The distributions and mean scores were determined and compared, and the correlations [Pearson (*r*)] and *p* values were assessed. The similarities and differences between the instruments were investigated regarding fatigue severity and the dimensions of fatigue. We also estimated the distance between the scores (standardized z-score) using multidimensional scaling (MDS) to visualize the similarities of the questions on the two-dimensional space. MDS statistically assesses and clusters similar questions using the single-linkage method of hierarchical agglomerative clustering (HAC). MDS helps clarify the structure and relations between questions [25]. Kruskal’s standardized residual sum of squares (STRESS) was also used to evaluate the goodness of fit of the instruments. The STRESS value ranges from 0 to 1, and close to 0 is considered appropriate for the results [26]. The MDS ALSCAL method in SPSS v. 20 was used for the statistical analysis.

## Results

### Characteristics of the participants

A total of 70 participants (median age 48, range 25–67), including 40 males (median age 50, range 32–66) and 30 females (median age 44, range 25–67), were included. They were grouped into three groups according to fatigue level: 20 were ‘mild’, 42 were ‘moderate’, and 8 were ‘severe’. The mean body mass index (BMI) was 25.2 ± 5.2. Age and BMI were higher in the ‘mild’ group than in the ‘severe’ group (age *p*  = 0.002, BMI *p*  = 0.06) (Table 1).

### Comparisons of the total scores of the MFI-K and mKCFQ according to fatigue level

**Fig. 1:**
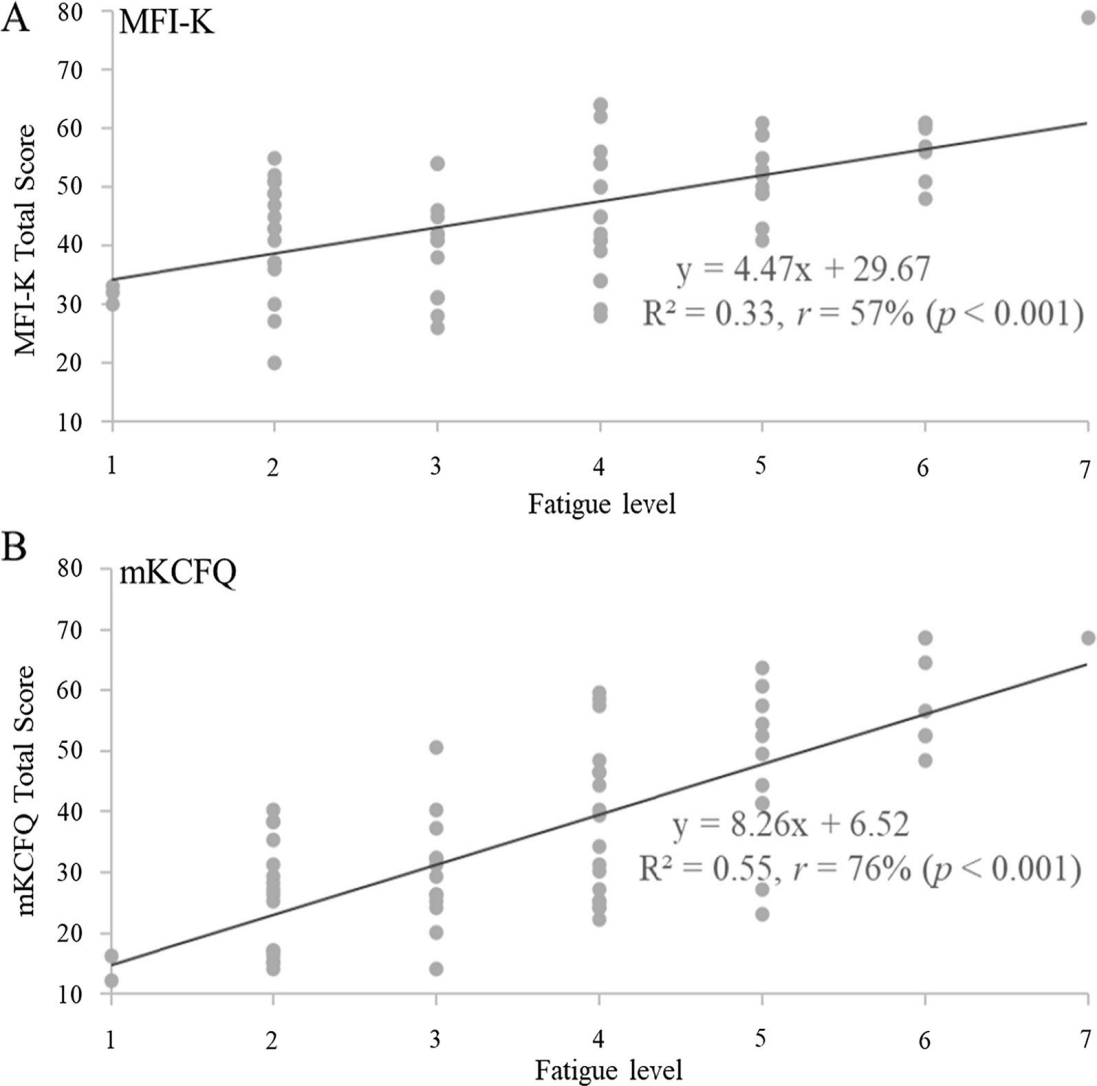
Scatter chart of the total scores of the MFI-K and mKCFQ according to fatigue level

**Fig. 2:**
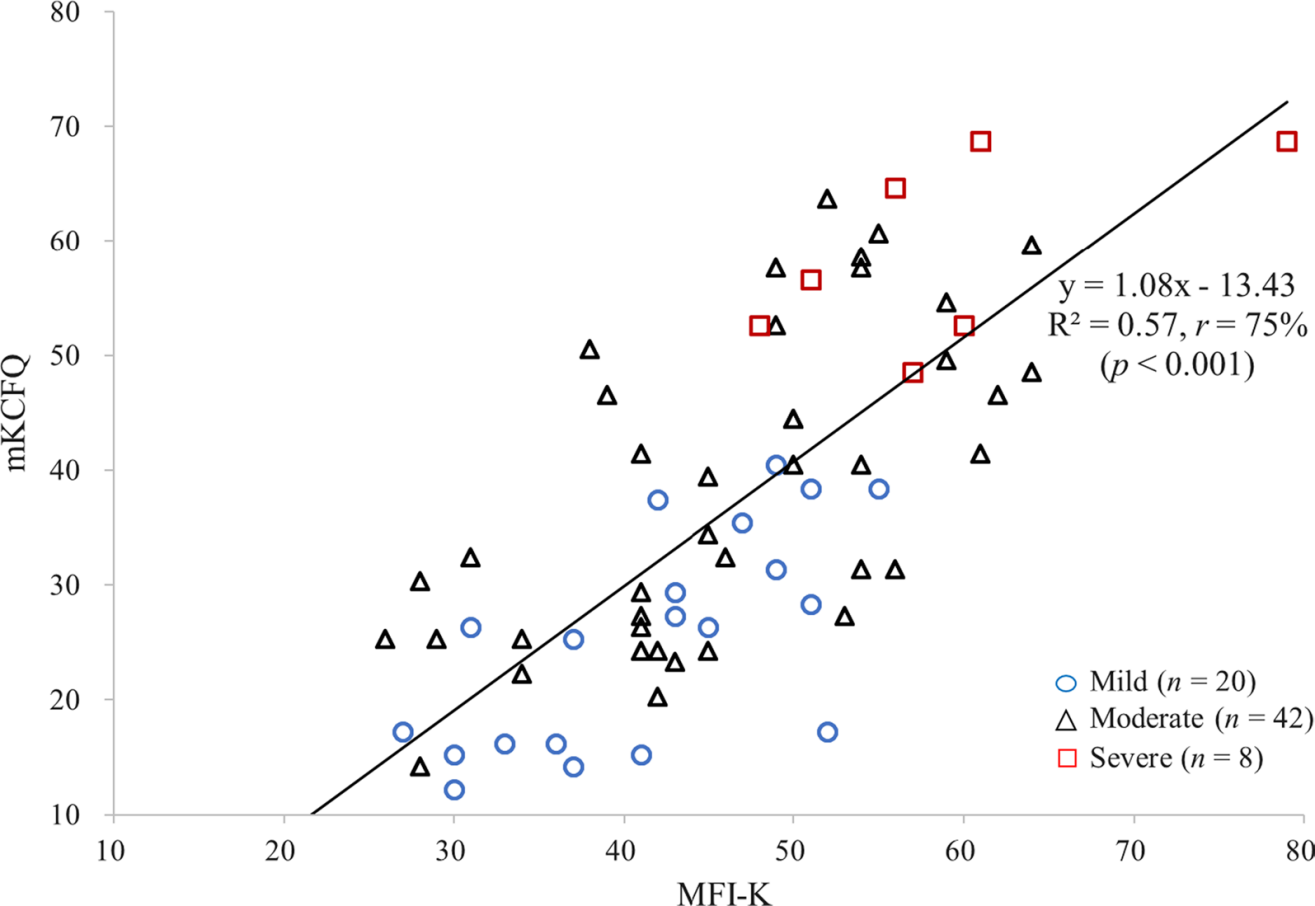
Scatter chart of the correlation between the total MFI-K score and the mKCFQ score

As expected, the total scores of both the MFI-K and mKCFQ showed a correlation with fatigue level (scored 1–7), while the correlation coefficients (*r*) was higher for the mKCFQ (*r*  = 0.76%, *p*  < 0.001) than for the MFI-K (*r*  = 0.57%, *p*  < 0.001). In addition, the mKCFQ score was more differentiated according to the fatigue level than the MFI-K score (mKCFQ: R^2^  = 0.55 vs. MFI-K: R^2^  = 0.33) (Fig. 1). Two instruments, the MFI-K and mKCFQ, also showed a good correlation, *r*  = 75% (*p*  < 0.001) (Fig. 2).

### Comparisons of MFI-K and mKCFQ according to the five dimensions of fatigue

The total MFI-K score (45.8 ± 11.3) was higher than the mKCFQ score (36.1 ± 16.2) for 70 participants. This pattern was found for both males (MFI-K 42.8 ± 11.1 vs. mKCFQ 31.5 ± 14.3) and females (49.8 ± 10.5 vs. 42.2 ± 16.8) and for both ‘total physical fatigue’ (28.1 ± 7.3 vs. 23.5 ± 10.6) and ‘total mental fatigue’ (17.7 ± 4.7 vs. 12.3 ± 6.9). In comparisons of the correlation coefficient (*r*) between the MFI-K and mKCFQ according to the five dimensions of fatigue, the ‘total physical fatigue’ score (*r*  = 76%, *p*  < 0.001) was highly correlated with the total ‘mental fatigue score’ (*r*  = 56%, *p * < 0.001). In particular, the scores of ‘general’ (*r*  = 79%) and ‘physical’ (*r*  = 66%) showed the highest correlations, while the lowest correlation was found for ‘reduced activity’ (*r*  = 26%). This pattern was also seen when we separately compared male and female participants (Table 2).

**Table 2:** Correlation (*r*) of MFI-K and mKCFQ in five dimensions (*n*  = 70)

Dimensions	Total (*n* = 70)			Male (*n* = 40)			Female (*n* = 30)		
	MFI-K	mKCFQ	*r*	MFI-K	mKCFQ	*r*	MFI-K	mKCFQ	*r*
Total physical fatigue	28.1 ± 7.3	23.5 ± 10.6	76***	26.5 ± 7.1	20.5 ± 9.7	73***	30.3 ± 6.9	26.6 ± 10.6	76***
General	10.4 ± 3.7	8.9 ± 4.3	79***	9.6 ± 3.8	7.7 ± 3.9	76***	11.3 ± 3.0	10.6 ± 4.2	82***
Physical	9.1 ± 3.0	7.8 ± 3.4	66***	8.4 ± 2.5	6.8 ± 3.0	56***	10.2 ± 3.2	9.1 ± 3.4	70***
Activity	8.6 ± 2.5	6.8 ± 4.1	26	8.5 ± 2.7	6.1 ± 4.1	22	8.8 ± 2.2	7.8 ± 3.9	33
Total mental fatigue	17.7 ± 4.7	12.3 ± 6.9	56***	16.3 ± 4.5	10.8 ± 5.9	56***	19.5 ± 4.4	14.6 ± 8.1	49**
Mental	8.7 ± 2.6	6.7 ± 3.6	56***	8.1 ± 3.3	6.1 ± 3.2	54***	9.7 ± 2.4	7.6 ± 3.9	53**
Motivation	8.9 ± 2.6	5.6 ± 3.6	41**	8.3 ± 2.6	4.7 ± 3.0	36*	9.8 ± 2.3	6.7 ± 3.9	38*
Total	45.8 ± 11.3	36.1 ± 16.2	75***	42.8 ± 11.1	31.5 ± 14.3	76***	49.8 ± 10.5	42.2 ± 16.8	70***

*r* Pearson correlation coefficient

*p  < 0.05, **p  < 0.01, ***p  < 0.001

### Comparisons of differentiating power of MFI-K and mKCFQ according to severity group

**Fig. 3:**
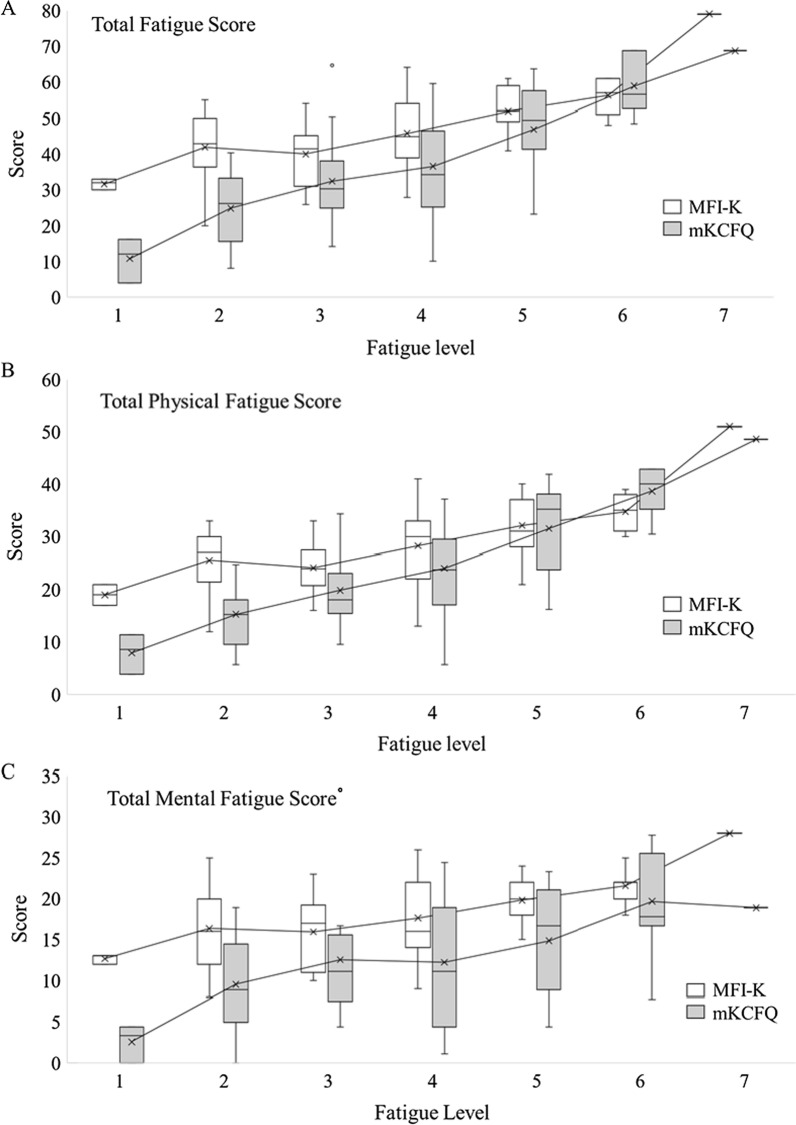
Box-Whisker plot of the MFI-K and mKCFQ scores according to fatigue level. According to the fatigue level assessed on a 7-point Likert scale, the total fatigue score (**A**), total physical fatigue score (**B**) and total mental fatigue score (**C**) of the MFI-K and mKCFQ are displayed

In Fig. 3a–c, the linear correlations of MFI-K and mKCFQ, according to fatigue level (Likert scale 1–7), was presented. However, the mKCFQ was likely to more distinctively differentiate the mild, moderate, and severe groups than the MFI-K. In detail, the difference in the total score between groups was larger for the mKCFQ (e.g., ‘Mild’–‘Moderate’  = 15.1, ‘Moderate’–‘Severe’  = 22.2) than for the MFI-K (e.g., ‘Mild’–‘Moderate’  = 5.4, and ‘Moderate’–‘Severe’  = 13.3). The score difference between the MFI-K and mKCFQ was larger in the group with a lower fatigue level (‘Mild’) (e.g., MFI-K 40.4–mKCFQ 22.8  = 17.6) but smaller in the group with severe fatigue (‘Severe’) (e.g., 59.1–60.1 = 1.0). These patterns were similarly repeated for the scores of ‘total physical fatigue’ and ‘total mental fatigue’ (Table 3; Fig. 3a–c). Moreover, the strongest correlation among the five dimensions was found for the ‘general’ score in the ‘severe group (*r*  = 91%, *p*  < 0.01).

**Table 3:** Comparisons of mean scores (±  SD) by severity group

Group	Items	Mild (*n* = 20)			Moderate (*n* = 42)			Severe (*n* = 8)		
		MFI-K	mKCFQ	*r* (%)	MFI-K	mKCFQ	*r* (%)	MFI-K	mKCFQ	*r* (%)
Total physical fatigue (score 60)	General	7.3 ± 2.5	5.0 ± 2.2	32	10.9 ± 2.8	9.7 ± 3.6	66***	15.4 ± 2.3	14.7 ± 2.6	91**
	Physical	8.0 ± 2.1	5.6 ± 2.7	41	9.2 ± 2.9	8.0 ± 3.0	72***	11.9 ± 3.8	12.3 ± 1.9	25
	Activity	9.4 ± 2.5	3.1 ± 2.5	65**	8.1 ± 2.5	7.4 ± 3.1	44**	9.5 ± 2.1	13.3 ± 2.4	19
	Total	24.6 ± 5.8	14.2 ± 6.1	79***	28.1 ± 6.8	24.6 ± 8.4	69***	36.8 ± 6.5	39.9 ± 5.5	52
Total mental fatigue (score 40)	Mental	7.4 ± 2.5	4.6 ± 2.8	57**	9.0 ± 2.4	7.0 ± 3.4	43**	11.1 ± 2.0	10.4 ± 2.5	21
	Motivation	8.5 ± 2.7	3.9 ± 2.9	39	8.7 ± 2.5	5.7 ± 3.3	32*	11.3 ± 1.4	9.2 ± 4.1	40
	Total	15.8 ± 4.7	8.5 ± 5.6	57**	17.6 ± 4.4	12.7 ± 6.4	42**	22.4 ± 3.0	19.6 ± 6.3	53
Total (score 100)		40.4 ± 9.7	22.8 ± 10.7	79***	45.8 ± 10.4	37.9 ± 13.2	65***	59.1 ± 9.3	60.1 ± 8.5	58

The *r* score and *p *value are rounded to the 2nd decimal

*p  < 0.05, **p  < 0.01, ***p  < 0.001

### Multidimensional scaling (MDS) analysis

**Fig. 4:**
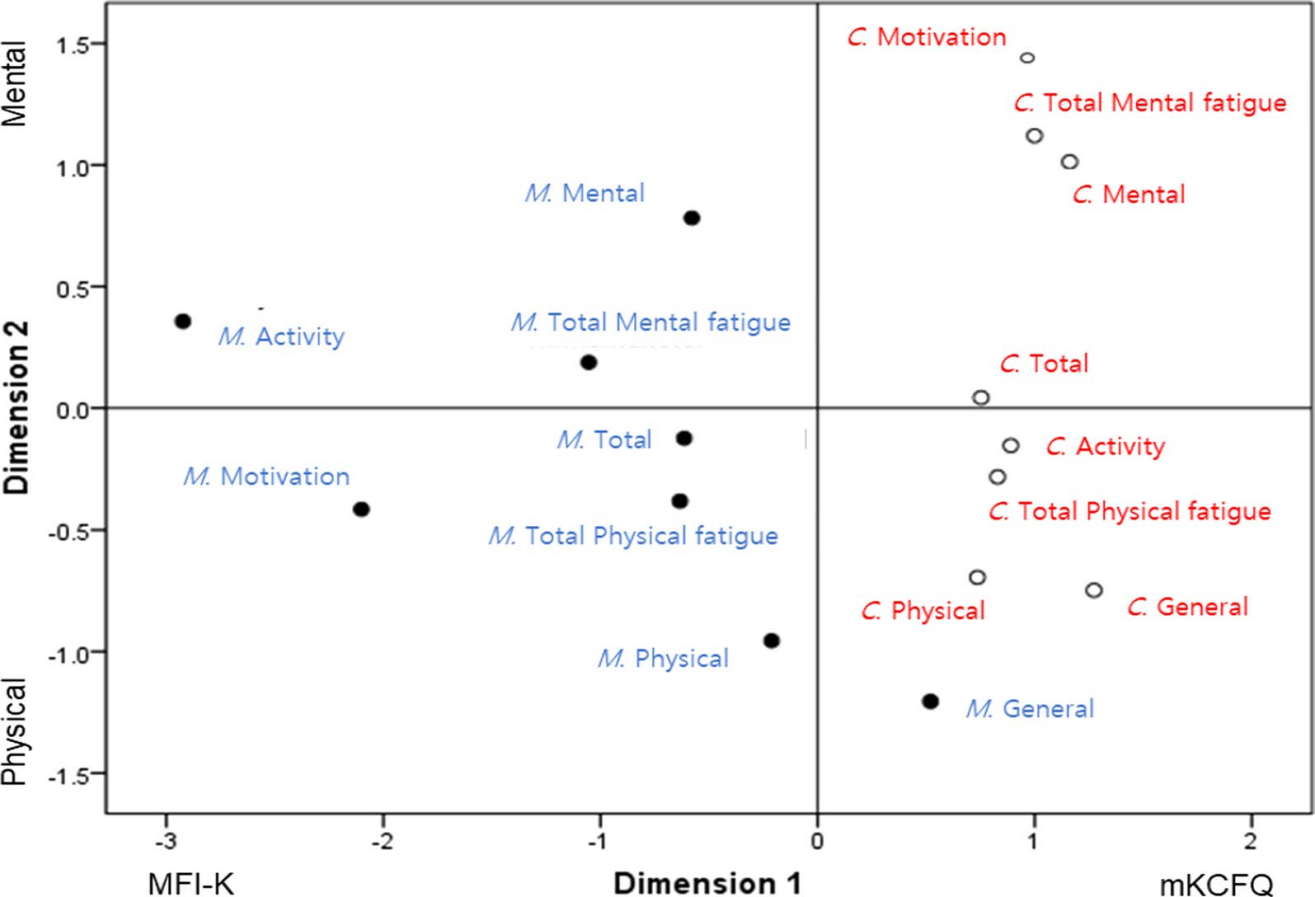
Euclidean distance model (EDM) of multidimensional scaling (MDS). *M* MFI-K; *C* mKCFQ

The distances of the scores between the MFI-K and mKCFQ were calculated using MDS analysis (Additional file 1: Table S2), and then they were structured on the two-dimensional Euclidean distance model (Fig. 4). The result clearly produced the separate components for the MFI-K and mKCFQ instruments (Dimension 1) and for ‘physical’ and ‘mental’ fatigue (Dimension 2), except the ‘general’ dimension of the MFI-K (M. General) and the ‘reduced activity’(M. Activity) and ‘motivation’ (M. Motivation) dimensions of the MFI-K, which were conversely located far away from the other dimensions. The result of STRESS was 0.118, which was considered to be fair and normal.

## Discussion

The CFQ and MFI were initially developed to measure the severity of various fatigue types, including CFS, in England in 1993 [14] and to measure cancer-related fatigue in the Netherlands in 1995 [15]. These two PRO-based instruments have been translated into as many languages and are commonly used for assessments of the extent and severity of fatigue in patient and nonpatient populations [27]. This study analyzed the similarities and differences of fatigue-assessment data from the Korean versions of MFI (MFI-K) and modified CFQ (mKCFQ) [22, 23] and aimed to produce important information regarding clinical choice and applications.

To evenly compare these instrument-derived fatigue scores, we converted mKCFQ (maximum 99 points) scores to 100 point-fitted scores to ensure that it was assessed on the same scale as the MFI-K score (maximum 100 points). As we expected, the total MFI-K and mKCFQ scores were significantly correlated (*r*  = 75%, *p*  < 0.001, Table 2; Fig. 2), but they showed some differences regarding severity groups and dimensions of fatigue for a single group comprising 70 participants. The MFI-K score was significantly higher than the mKCFQ score by 1.3-fold for 70 participants (45.8 ± 11.3 vs. 36.1 ± 16.2, *p*  < 0.001), 40 male and 30 female participants (Table 2). This high-score result in MFI-K mainly came from the score difference (17.6 points) for the ‘mild’-fatigue group (40.4 ± 9.7 vs. 22.8 ± 10.7, Table 3), which is shown clearly in the Box-Whisker plot (Fig. 3a). These results may explain the possibility of a tendency of MFI-K to be scored high, especially for the general population with a very low level of fatigue. In fact, this pattern of the MFI-K tool was seen in its initial validation study, which showed the comparison between the MFI-K total score (maximum 100) and VAS (maximum 100). In this study, compared to the ‘severe’ (5.5 points) and ‘moderate’ (19.9 points) groups, the ‘mild’ fatigue group (24.5 points) had the largest difference [22]. This can be due to MFI-K having more number of questions than mKCFQ, thus MFI-K may be sensitive to certain aspects of fatigue questions.

In general, fatigue is grouped into two categories, so-called physical and mental fatigue, in most studies [28, 29]. The two instruments included in this study are also structured to distinguish between physical and mental fatigue assessments, and the responses to physical-mental fatigue-focusing questions were well differentiated from each other [22, 23, 30]. When we analyzed the correlation between the MFI-K and mKCFQ in the aspect of the physical and mental fatigue categories, each of the ‘physical’ and ‘mental’ fatigue scores correlated well between the two instruments, while the physical fatigue dimensions (‘general’, ‘physical’, and ‘activity’) were more highly correlated than the mental fatigue dimensions (‘mental’ and ‘motivation’, Table 2). We confirmed this feature in both box-whisker plots (Fig. 3b, c) and EDM analysis (Fig. 4). This would reflect the similarity of ‘physical’ fatigue assessments between two instruments but further dissimilarity of ‘mental’ fatigue assessments. In fact, previous studies using MFI reported similar results, showing a higher correlation of ‘general’ and ‘physical’ fatigue scores with VAS-based overall fatigue levels than scores of ‘activity’ and ‘motivation’ [15, 31]. These results may mean that the mKCFQ further sharply differentiates ‘mental’ fatigue severity from low-to-severe levels compared to the MFI-K.

Among the three fatigue groups and five dimensions, ‘general’ fatigue (*r*  = 91%, MFI-K vs. mKCFQ  = 15.4 ± 2.3 vs. 14.7 ± 2.6) and ‘reduced activity’ (*r*  = 19%, MFI-K vs. mKCFQ  = 9.5 ± 2.1 vs. 13.3 ± 2.4) showed the highest and lowest correlations in the ‘severe’ fatigue group (Table 3). One possible reason might be the misunderstanding of questions such as “I feel very active”, “I think I do a lot in a day”, and “I feel like doing all sorts of nice things” in the MFI-K, which may seem less likely related to fatigue-related symptoms but rather more general-behavioral questions (Additional file 1: Table S1). This is reflected in the two-dimensional matrix in Fig. 4, which, unlike the others, reduced ‘activity’ in the mental dimension (Fig. 4). From the results above, we suspect that the questions for reduced ‘activity’ and ‘motivation’ in MFI-K possibly misled the responses. In fact, a study of the reliability and validity of the MFI-K using outpatients of the Department of Family Medicine showed very low correlations with the VAS (‘reduced activity’ 0.087, and ‘motivation’ 0.159) [22]. The present study showed a relatively better ability of the mKCFQ to appropriately assess the severity of fatigue. In fact, CFQ has been criticized as an operational method that asks one to choose among “less than usual”, “no more than usual”, “more than usual”, and “much more than usual”, which may lead the answers to the extreme end of the scale [32]. Extreme scoring is likely to cause an inability to discriminate between different groups [32]. Thus, the mKCFQ was designed on a 10-point Likert scale, which allows for the assessment of small fatigue differences among participants and changes after therapeutic interventions [21, 23].

In summary, the MFI-K and mKCFQ are likely to be sensitive in discriminating the ‘severe’ fatigue group, while the mKCFQ seems to be more suitable for participants with low levels of fatigue symptoms. In addition, clinicians or researchers may need to be aware of the low sensitivity of ‘reduced activity’ and ‘motivation’ relative to ‘general’ and ‘physical’ fatigue. For a certain illness such as CFS, the optimal fatigue scale should accurately identify specifications with multiple dimensions of fatigue in the process of both the diagnosis and assessment of therapeutics [33]. The MFI and CFQ and their Korean versions (MFI-K and mKCFQ) have been adapted in clinics and clinical trials for CFS patients [21, 23]; however, we still need to increase the specificity to differentiate patients by adding specific fatigue dimensions for postexertion malaise (PEM), one of the primary symptoms in patients with CFS [33]. This study has some limitations, such as a relatively small number of participants, especially for the ‘severe’ fatigue group and the inclusion of exclusively university personnel. This study was performed in Korea using Korean versions of fatigue scales; thus, limits in generalization with other languages. The correlations of the two instruments were compared based on the scores of the total and each domain. Further studies are needed among larger-scale populations with diverse fatigue severities.

## Conclusions

We compared the Korean version of the MFI (MFI-K) and modified CFQ (mKCFQ) among a group of university personnel and analyzed the similarities and differences using correlations and the MDS. Overall, MFI-K and mKCFQ were highly correlated, while mKCFQ discriminated the severity of fatigue in a wider spectrum than MFI-K. Both instruments were more correlated for ‘physical’ symptoms than for reduced ‘activity’ and ‘motivation’. Further research is required to improve those dimensions to identify fatigue patients with multiple dimensions of fatigue, such as CFS.

## Supplementary Information

**Additional file 1: Table S1. **Classification of the questions in sections for MFI-K and mKCFQ. **Table S2. **Distance matrix.

## Data Availability

The data analyzed in this study are available from the first author on request.

## Abbreviations

MFI-K: Korean version of multidimensional fatigue inventory
mKCFQ: Korean version of modified chalder fatigue scale
CFS: Chronic fatigue syndrome
MDS: Multidimensional scaling
EDM: Euclidean distance model
