# Supplementary material for: Comparison of assessment scores for fatigue between multidimensional fatigue inventory (MFI-K) and modified chalder fatigue scale (mKCFQ)
Source: J Transl Med. 2022 Jan 3;20:8. doi: 10.1186/s12967-021-03219-0 (PMC8722196; doi:10.1186/s12967-021-03219-0)
Supplement: Supplementary file 1 — Additional file 1: Table S1. Classification of the questions in sections for MFI-K and mKCFQ. Table S2. Distance matrix. [file 12967_2021_3219_MOESM1_ESM.docx]

**Supplementary Table 1.** Classification of the questions in sections of MFI-K and mKCFQ

| 구분 (Items) | | MFI-K (20 Questions, 5 point scale, total score 100) | mKCFQ (11 Questions, 9 point scale, total score 99) |
| --- | --- | --- | --- |
| 육체적 피로  (Physical fatigue total) | 전반적 피로  (General fatigue) | 1. 나는 몸 상태가 좋다. (I feel fit.)  5. 나는 피곤함을 느낀다. (I feel tired.)  12. 나는 가뿐하다. (I am rested.)  16. 나는 쉽게 피곤해진다. (I tire easily.)  총점 20점 (Total score: 20) | 1. 당신이 평소 느끼는 피로의 정도는 어떻습니까?  (Do you have problems with tiredness?)  2 당신은 어느 정도의 휴식이 필요합니까? (Do you need to rest more?)  총점 18점 (Total score: 18) |
|  | 활동성 저하 (Reduced activity) | 3. 나는 매우 활동적이라고 생각한다. (I feel very active.)  6. 나는 하루 동안에 아주 많은 일을 해낸다고 생각한다.  (I think I do a lot in a day.)  10. 나는 하루 동안에 아주 적은 일을 한다고 생각한다.  (I think I do very little in a day.)  17. 나는 처리한 일이 거의 없다. (I get little done.) 20점 | 3. 당신은 어느 정도의 졸음을 느끼십니까? (Do you feel sleepy or drowsy?)  4. 당신은 피로감 때문에 일을 시작할 때 힘이 듭니까?  (Do you have problems starting things?)  18점 |
|  | 육체적 피로  (Physical fatigue) | 1. 육체적으로 나는 아주 가벼운 일 밖에 할 수 없다.  (Physically I feel only able to do a little.)  8. 육체적으로 나는 많은 일을 해낼 수 있다.  (Physically I can take on a lot.)  14. 육체적으로 나는 몸 상태가 나쁘다고 생각한다.  (Physically I feel I am in a bad condition.)  20. 육체적으로 나는 몸 상태가 아주 좋다고 생각한다.  (Physically I feel I am in an excellent condition.) 20점 | 5. 당신은 기운(기력)이 없다고 느끼십니까? (Do you lack energy?)  6. 당신은 근육의 힘이 약해졌다고 느끼십니까?  (Do you have less strength in your muscles?)  7. 당신은 허약해졌다고 느끼십니까? (Do you feel weak?)  27점 |
| 정신적 피로 (Mental fatigue total) | 정신적 피로  (Mental fatigue) | 7. 나는 어떤 일을 하는 동안 그 일에 대한 생각을 계속  유지할 수 있다.  (When I am doing something, I can keep my thoughts on it.)  11. 나는 집중을 잘 할 수 있다. (I can concentrate well.)  13. 어떤 일에 집중하기 위해서 많은 노력이 필요하다.  (It takes a lot of effort to concentrate on things.)  19. 생각이 쉽게 산란해진다. (My thoughts easily wander.) 20점 | 8. 당신은 일에 대한 집중력이 떨어졌습니까?  (Do you have difficulties concentrating?)  11. 당신의 기억력 저하는 없습니까? (How is your memory?)  18점 |
|  | 동기유발 저하  (Reduced motivation) | 4. 나는 온갖 흥미로운 일들에 빠져들기를 좋아한다.  (I feel like doing all sorts of nice things.)  9. 나는 어떤 일을 하는 것이 염려스럽다.  (I dread having to do things.)  15. 나는 계획하고 있는 일들이 많다. (I have a lot of plans.)  18. 나는 어떠한 일도 하고 싶지 않다.  (I don’t feel like doing anything.) 20점 | 9. 당신은 명료하게 생각하는 것에 어려움이 있습니까?  (Do you make slips of the tongue when speaking?)  10. 당신은 말할 때 적절한 단어 선정이 어려운 경우가 있습니까?  (Do you find it more difficult to find the right word?)  18점 |

MFI-K, Korean version of multidimensional inventory. mKCFQ, Korean version of modified Chalder fatigue scale.

**Supplementary Table 2.** Distance matrix^†^

|  | | **MFI-K** | | | | | | | | **mKCFQ** | | | | | | | |
| --- | --- | --- | --- | --- | --- | --- | --- | --- | --- | --- | --- | --- | --- | --- | --- | --- | --- |
|  |  | G | P | M | A | Mot | Pt | Mt | Total | G | P | M | A | Mot | Pt | Mt | Total |
| **MFI-K** | G | 0 |  |  |  |  |  |  |  |  |  |  |  |  |  |  |  |
|  | P | **1.23** | 0 |  |  |  |  |  |  |  |  |  |  |  |  |  |  |
|  | M | 1.83 | 1.83 | 0 |  |  |  |  |  |  |  |  |  |  |  |  |  |
|  | A | 3.89 | 2.82 | 2.82 | 0 |  |  |  |  |  |  |  |  |  |  |  |  |
|  | Mot | 2.82 | 2.38 | 1.83 | 1.83 | 0 |  |  |  |  |  |  |  |  |  |  |  |
|  | Pt | 1.12 | **0.71** | 1.18 | 1.83 | 1.83 | 0 |  |  |  |  |  |  |  |  |  |  |
|  | Mt | 1.83 | 1.83 | **0.71** | 1.88 | **0.71** | 1.18 | 0 |  |  |  |  |  |  |  |  |  |
|  | Total | 1.18 | 1.18 | 0.97 | 1.83 | 1.18 | **0.27** | 0.59 | 0 |  |  |  |  |  |  |  |  |
| **mKCFQ** | G | **1.18** | 1.83 | 2.27 | 4.34 | 3.39 | 1.83 | 2.27 | 1.83 | 0 |  |  |  |  |  |  |  |
|  | P | **1.18** | 1.31 | 2.27 | 3.70 | 2.82 | **1.18** | 2.27 | 1.23 | 1.18 | 0 |  |  |  |  |  |  |
|  | M | 2.30 | 2.38 | 1.90 | 4.14 | 3.51 | 2.27 | 2.27 | 1.90 | 1.83 | 1.83 | 0 |  |  |  |  |  |
|  | A | **1.18** | 1.83 | 1.83 | 3.89 | 2.82 | 1.54 | 1.83 | 1.31 | 1.12 | 1.18 | 1.29 | 0 |  |  |  |  |
|  | Mot | 2.82 | 2.38 | 1.90 | 3.89 | 3.70 | 2.38 | 2.38 | 2.27 | 2.27 | 1.83 | **0.71** | 1.83 | 0 |  |  |  |
|  | Pt | **1.18** | 1.29 | 1.83 | 3.89 | 2.82 | **1.18** | 1.83 | **1.18** | 0.59 | 0.71 | 1.23 | **0.27** | 1.83 | 0 |  |  |
|  | Mt | 2.38 | 2.27 | 1.83 | 3.89 | 3.51 | 2.27 | 2.27 | 1.90 | 1.89 | 1.83 | **0.27** | 1.29 | **0.27** | 1.29 | 0 |  |
|  | Total | **1.18** | 1.31 | 1.83 | 3.70 | 2.82 | **1.18** | 1.83 | **1.18** | 0.97 | 0.94 | 0.97 | 0.71 | 1.18 | **0.27** | 0.94 | 0 |
|  | | | | | | | | | | | | | | | | | |

MFI-K, Korean version of multidimensional inventory. mKCFQ, Korean version of modified Chalder fatigue scale. G, general. P, physical. M, mental. A, reduced activity. Mot, reduced motivation. Pt, Physical total. Mt, Mental total. Bolded number, the smallest number in each section. ^†^, optimally scaled data of the compared data sets.
